# Are caveolin-1 minor alleles more likely to be risk alleles in insulin resistance mechanisms in metabolic diseases?

**DOI:** 10.1186/s13104-021-05597-6

**Published:** 2021-05-17

**Authors:** Faezeh Abaj, Said Abdul Ghafour Saeedy, Khadijeh Mirzaei

**Affiliations:** 1 Department of Community Nutrition, School of Nutritional Sciences and Dietetics, Tehran University of Medical Sciences (TUMS), No. 44, Hojjat-dost Alley, Naderi St., Keshavarz Blvd, P.O. Box, 14155-6117 Tehran, Iran; 2 Department of Paraclinic, School of Medicine, Herat University, Herat, Afghanistan

**Keywords:** Caveolin-1, Insulin resistance, Dyslipidemia, Polymorphism, Obesity

## Abstract

**Objectives:**

Obesity and insulin resistance (IR) are interrelated in a range of ways. The IR-obesity relationship is not a cause-and-effect association. Molecular biology research has made tremendous strides in discovering contributors to find this association. Genes that control adipocyte function such as caveolin-1 (*CAV1*)*;* probably interact in the pathogenesis of human IR in this context. The involvement of *CAV1* in glucose/lipid homeostasis is revealed and could modify the signaling of the insulin receptor. We examined the association between *CAV1* and insulin signaling in modifying dyslipidemia and fat composition in overweight and obese women with a prevalent variant in the *CAV1* gene.

**Results:**

Minor allele carriers were slightly older and had higher BMI (p = 0.02), FMI (p = 0.006), and VLF (p = 0.01) values; and tended to have lower total cholesterol TC (p = 0.04), low-density lipoprotein cholesterol (LDL-C) (p = 0.001) and high-density lipoprotein cholesterol (HDL-C) (p = 0.003). HOMA-IR levels predicted fat mass index (FMI) 0.47 (0.08, 0.87), visceral fat level (VFL) 0.65 (0.23, 1.07), TC 6.82 (1.76, 11.88) and HDL-C − 1.663 (− 3.11, − 0.214) only between minor allele carriers in adjusted models. (β, CI). Our results cast a new light on the IR mechanism and future studies will elucidate the clinical relevance of *CAV1*-IR in patients with dyslipidemia and high fat composition.

## Introduction

Preceding studies have established the principal role of Insulin resistance (IR) and resultant hyperinsulinemia in cardiometabolic risk factors [1, 2]. Inappropriate signaling of insulin has been associated with impaired fat distribution, adipocyte metabolism, and dyslipidemia [3]. According to studies, IR is associated with obesity, especially visceral obesity, hypercholesterolemia, hypertriglyceridemia, and low HDL-C concentration [4–7]. Obesity and IR are interrelated in various forms [8]. Obesity-associated IR is the main risk factor for type 2 diabetes and cardiovascular disease [9, 10]. Over the past years, a vast range of endocrine, inflammatory, neural, and cell-intrinsic paths have been revealed to be dysregulated in obesity. While it is likely that one of these factors plays a principal role, many of these factors are inter-reliant, and their interaction probably underlies the pathophysiology of IR [11]. Indeed, the relationship between obesity and IR is not possible a cause-and-effect relationship. Molecular biology research has made wonderful strides in discovering and defining many more contributors to find this relationship. The exact mechanisms for the insulin-induced defects including adipocyte dysfunction are unclear. However, several genomic and nongenomic pathways are present that mediate these IR effects. Furthermore, these effects could be secondary to alternative plasma membrane proteins, and other receptors [12].

Caveolin-1 (CAV1), a 21–24 kDa integral membrane protein, is the main structural protein of caveolae. The location of the *CAV1* gene is on human chromosome 7 (7q31.1) and it contains 3 exons that select intronic SNPs. CAV1 acts as scaffolding and has been involved in transmembrane signaling [13]. CAV1 is an important constituent of the lipid raft that controls their activity and cooperates with several signaling pathways, involving steroid receptors [14]. Rs3807992 is a mutation located in intron 2 of the *CAV1* with the substitute of A to G. The functional study at the mRNA and protein levels for the function of *CAV1*(3807992) is still deficient. In literature, the source of most of the evidence is from studies in other SNPs in this gene. For example, the minor *CAV1* rs926198 allele is linked with lower CAV1 expression levels [15]. Two common polymorphisms in the *CAV1* gene are rs3807989 and rs1049334, and both of them are described to be considerably coupled with elevated expression of CAV1 mRNA and protein [16, 17]. The association between metabolic disease and *CAV1* deficiency has been studied mainly in animal models. *CAV1* knockout mice display numerous metabolic defects, including hyperglycemia, IR, and dyslipidemia, like those seen in humans with severe, nonsense *CAV1* mutations [18–20]. As has been explored in preceding studies, *CAV1* gene variants were correlated with IR, dyslipidemia, diabetes mellitus, and metabolic syndrome [21].

The main location of the insulin receptor of the adipocyte is suggested to be in caveolae and bound to immobilized caveolin to excites their signaling [22]. The clinical significance of the relationship between *CAV1* and IR-mediated mechanisms in adipose tissue in the pathogenesis of IR in humans has been discussed. Here, we talk about the hypothesis that the *CAV*1-IR mechanism is a mediator of cardiometabolic disorder in caveolin genotypes.

Despite the developing knowledge in understanding the role of insulin pathways in dyslipidemia, to date no study has considered whether this mechanism works the same in all participants; and whether minor alleles are more likely to be risk alleles in IR mechanisms in metabolic diseases or not. We investigated the potential interplay between IR levels and a selected human *CAV1* gene variant (rs3807992) in modulating dyslipidemia and body fat composition.

## Main text

### Method

#### Subjects

For this cross-sectional study, we analyzed the data which was collected from samples of Tehranian overweight/obese females, aged over 18 and were before menopause. Women with a history of chronic and inflammatory disease and who were pregnant or lactating, taking any therapeutic medications, or follow a special diet or supplements were excluded. After the final exclusion, 404 women remained in the present analysis. The study participants were fully informed concerning the research protocol and they signed a consent form before taking part in the research. Tehran University of medical sciences (TUMS) ethics committee agreed with these protocols (97-03-161-41017).

#### Procedures

Anthropometric variables were measured by standard protocols. We used digital scales for measuring weight, and measuring tape for measurement of height and waist circumference while the subjects were standing with bare feet. Body mass index (BMI) was calculated as a ratio of weight (kg) to height in meters squared. Bioelectrical impedance analysis [BIA 770 (South Korea)] is an electrical method of assessing human body composition and was used to assess the VFL, body fat mass (BFM), and FMI. International Physical Activity Questionnaires (IPAQ) were used to assess physical activity [23]. A Food Frequency Questionnaire (FFQ) was used to calculate energy intake.

#### Genotyping

The Mini Columns kit (Type G; Genall; Exgene) was used for DNA extraction. The *CAV1* SNP (rs3807992) was genotyped by PCR–RFLP method, using primers, Forward: 3′AGTATTGACCTGATTTGCCATG5′ Reverse: 5′GTCTTCTGGAAAAAGCACATGA-3′. According to our previous study [24].

#### Measurements of biochemical parameters

We collected blood samples after 10–12 h of overnight fasting. For measurement of FPG, glucose oxidase phenol 4-aminoantipyrine peroxidase (GOD/PAP) method was used [25]. Furthermore, triacylglycerol kits (Pars Azmoon Inc, Tehran, Iran) were applied for the determination of serum TG level. The total TC level was evaluated by the cholesterol oxidase phenol 4-aminoantipyrine peroxidase (CHOD-PAP) method [26]. Besides, high-density lipoprotein (HDL) and low-density lipoprotein (LDL) were measured by the direct method and immunoinhibition [27].

#### HOMA-IR calculation

Homeostatic model assessment (HOMA) calculated by using this formula: HOMA-IR = ¼ [FPG (mmol/l) × fasting plasma insulin (mIU/l)]/22.5 [28].

### Statistical analysis

IBM SPSS statistics (version 25) was used for all phases of the analysis (SPSS Inc, Chicago, IL, USA). The evaluation of the normality of quantitative variables was conducted by K–S (Kolmogorov–Smirnov) test. Independent Student’s *t*-test was used to baseline analysis comparing by genotype status. The adjusted linear regression model was used to assess the relationship between HOMA-IR levels and cardiometabolic variables, first, in all participants without grouping; and subsequently in whom that were grouped by rs387992 genotype status adjusted for age, energy intake, and IPAC index. Data are presented as means ± SD, and *P* < *0.05* is considered statistically significant.

## Results

### Study population

A total of 404 adults were analyzed. Our study had the following characteristics: age 36.67 ± 9.1 years, body mass index (kg/m^2^) of 31.26 ± 4.2. Using a dominant model for genetic analysis, a homozygous major allele (GG) of the rs3807992 *CAV1* variant was observed in 50% of participants, and the other 50% were minor allele carriers (23.31% had an AG genotype and 26.6% had an AA genotype).

### Clinical and biochemical characteristics categorized by CAV1 genotype:

**Fig. 1:**
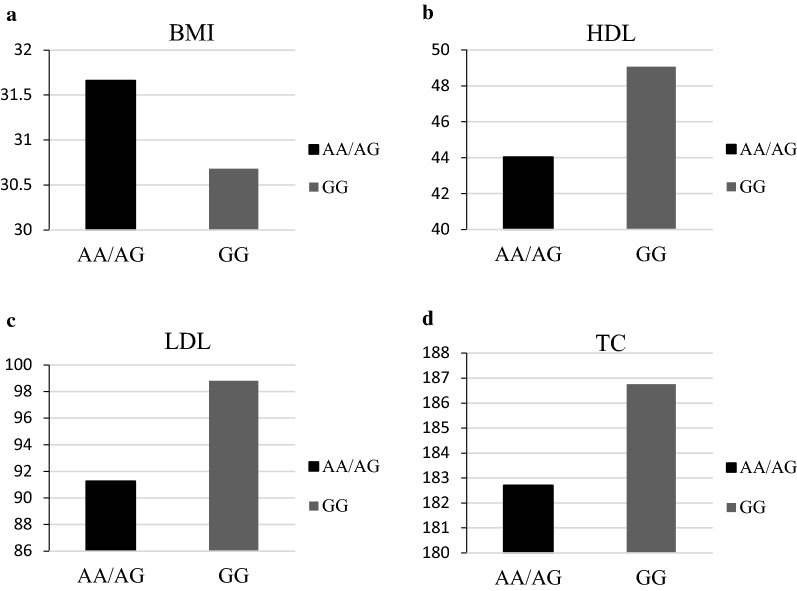
Clinical and biochemical characteristics categorized by Cav-1 variant rs3807992. **a** BMI. **b** HDL. **c** LDL. **d** TC

Compared with those with the homozygous major allele of rs3807992 *CAV1* variant, minor allele carriers had no statistical differences (*p* > *0.05*) in FPG, plasma insulin, and HOMA-IR, as are described in Table 1. Minor allele carriers were slightly older (*p* = *0.05*) and had higher BMI (*p* = *0.02*), FMI (*p* = *0.006*), VLF (*p* = *0.01*) values, and tended to have lower TC (*p* = *0.04*), LDL-C (*p* = *0.001*), and HDL-C levels (*p* = *0.003*) (Fig. 1).

**Table 1:** Clinical and biochemical characteristics categorized by Cav-1 variant rs3807992

Variable	Minor allele carrier AA/AG	Major allele carrier GG	P-value*
Age	35.75 ± 8.78	37.56 ± 9.49	0.05
BMI	31.66 ± 4.46	30.68 ± 4.01	**0.02**
FMI	3.46 ± 13.83	3.25 ± 12.93	**0.006**
VFL	3.11 ± 16.28	3.40 ± 15.46	**0.01**
FBS	86.95 ± 9.75	87.98 ± 9.62	0.31
Insulin	1.22 ± 0.25	1.21 ± 0.22	0.61
HOMA-IR	3.53 ± 1.82	3.27 ± 1.21	0.54
TG	133.31 ± 84.14	113.11 ± 51.20	0.14
HDL	44.04 ± 10.16	49.07 ± 11.16	**0.003**
LDL	91.27 ± 25.07	98.80 ± 22.66	**0.001**
TC	182.71 ± 37.36	186.76 ± 33.74	**0.04**

A significant P-values are indicated in bold (significance considered P < 0.05)

Values are mean (SD)

*Adjusted model by age, energy intake, IPAC and BMI

### The CAV1 variant modulates the effect of HOMA-IR on lipid hemostasis and lipid profile

A statistically significant relationship was found between *CAV1* variants and anthropometric data and lipid profile values. Since rs387992 genotype and HOMA-IR levels were associated with anthropometric data and lipid profile values, we considered whether they were independent predictors in a linear regression model or not.

As shown in Table 2, we found both rs3807992 status and HOMA-IR levels significant predictors of lipid profiles and anthropometric status with a statistically significant interaction. To further analyze the relationship between these predictors, we stratified the effect of HOMA-IR on TC by genotype. Interestingly, HOMA-IR levels predicted TC between all participant 5.32 (153.65, 178.56) (*p* = *0.003*) and only in minor allele carriers 6.82 (1.76, 11.88) (*p* = *0.009*) in adjusted models. Also, lower HDL-C levels were associated with HOMA-IR levels in all participants-1.473 (− 2.52, − 0.42) (*p* = *0.006*). Moreover, these results were driven by the effect on minor allele carriers of the *CAV1* gene variant. There was a consistent significant interaction when analyzing HOMA-IR levels with rs3807992 genotypes predicting HDL-C levels − 1.663 (− 3.11, − 0.214) (*p* = *0.02*).

**Table 2:** Adjusted linear regression models assessing the relationship between HOMA-IR levels and cardiometabolic variables in all participants by rs387992 genotype status

Cardiometabolic variable	Category^a^	HOMA-IR Adjusted b^b^ (95% CI) P value
FMI	All participants	0.449 (0.17, 0.72) **P = 0.002**
	Minor allele carriers	0.47 (0.08, 0.87) **P = 0.01**
	Major allele homozygous	0.37 (− 0.037, 0.77) *P* = 0.07
VFL	All participants	0.51 (0.19, 0.83) **P = 0.002**
	Minor allele carriers	0.65 (0.23, 1.07) **P = 0.003**
	Major allele homozygous	0.32 (− 0.18, 0.83) *P* = 0.21
TG	All participants	15.25 (10.88, 21.63) *P* ≤ 0.0001
	Minor allele carriers	17.21 (8.55, 25.88) *P* ≤ 0.0001
	Major allele homozygous	17.6 (10.40, 24.81) *P* ≤ 0.0001
HDL	All participants	− 1.473 (− 2.52, − 0.42) ***P* = 0.006**
	Minor allele carriers	− 1.663 (− 3.11, − 0.214) ***P* = 0.02**
	Major allele homozygous	− 0.87 (− 2.5, 0.75) *P* = 0.29
LDL	All participants	1.64 (− 0.72, 4.01) *P* = 0.17
	Minor allele carriers	2.47 (− 0.095, 5.9) *P* = 0.15
	Major allele homozygous	1.13 (− 2.27, 4.54) *P* = 0.51
TC	All participants	5.32 (153.65, 178.56) ***P* = 0.003**
	Minor allele carriers	6.82 (1.76, 11.88) **P = 0.009**
	Major allele homozygous	3.88 (− 1.18, 8.95) *P* = 0.13

A significant P-values are indicated in bold (significance considered P < 0.05

HOMA-IR indicates homeostasis model assessment of insulin resistance

^a^Categorized by Caveolin 1 genotype rs926198

^b^Linear regression adjusted by age, energy intake, physical activity level

We assessed whether changes in HOMA-IR levels predicted changes in VLF and FMI levels. Indeed, HOMA-IR predicted higher VLF between all participants 0.51 (0.19, 0.83) (*p* = *0.002*) and in *CAV1* minor allele carriers 0.65 (0.23, 1.07) (*p* = *0.003*) but not in major allele homozygotes. Moreover, higher FMI levels were associated with HOMA-IR levels in adjusted models in all participants 0.449 (0.17, 0.72) (*P* = *0.002*), but again, these results were driven by the effect on minor allele carriers of the *CAV1* gene variant 0.47 (0.08, 0.87) (*P* = *0.01*) but not in major allele homozygotes. In contrast, HOMA-IR levels were not associated with TG and LDL-C, because they were similar in both *CAV1* genotype groups.

## Discussion

The unfolding story of caveolae and caveolin signaling in human health has started in the past. Our study has shown that the insulin pathway mediates some of the metabolic characteristics associated with defective caveolin genotype. Although HOMA-IR levels were similar in both carriers and non-carriers of the *CAV1* minor allele, this study showed that HOMA-IR significantly estimates changes in TC, HDL-C, VLF, and FMI levels only in minor allele carriers of *CAV1* gene variant. The different effect of HOMA-IR on VLF, FMI, HDL-C, and TC levels in minor allele carriers may be attributed to an insulin signaling defect predicted by *CAV1* genotype status. The relationship between TG and HOMA-IR seems to indicate that the effects of *CAV1* on TG are IR independent.

The relationship of HOMA-IR levels activation with higher TC, lower HDL-C, and alteration in lipid composition in participants is supported by different studies [7, 12, 29–31]. IR is related to CVD and T2DM risk factors, such as dyslipidemia and obesity (mainly visceral obesity). Obesity-IR association has been investigated previously, some studies suggested obesity as the cause of IR since human and animal studies show that weight loss/gain correlates closely with increasing/decreasing insulin sensitivity, respectively [32–34]. In contrast, several epidemiologic studies have found that one-third of obese individuals fall into the category of “metabolically healthy obese”, they have additional body fat but no metabolic disruptions that describe the symptoms of IR [35]. In terms of this hypothesis, some studies of healthy people nourished with controlled high- and low-fat diets have shown that low-fat diets cause noteworthy improvements in whole-body insulin sensitivity [36–40]. In all these interventions, body weight was maintained unchanged; thus, corrections in insulin sensitivity on low-fat diets were not described by variations in weight. Though practically, obesity may obscure the association between fat consumption and IR. Clinical trials reveal that excessive levels of dietary fat can worsen insulin sensitivity independent of body weight variations [41]. On the other side, some researchers have suggested that IR is a “defense mechanism” of obese people against further weight gain [41].

There are two portal systems in the body: hypothalamus-pituitary and pancreas-liver. If the problem starts in the liver, then the pancreas has to secrete more insulin by mass action to make the liver do its job; this raises insulin levels all over the body, promotes adipogenesis, and generates peripheral IR all at the same time. Instead, if the IR starts first in the hypothalamus, the leptin signal is also antagonized there, causes increased appetite and weight gain and finally peripheral IR [42, 43]. Experimental studies indicate that it is a 2-way street. At first, IR can develop in the liver or central nervous system. But unfortunately, it is not possible to determine the location of this onset. Following the onset of IR in one of these two areas, hyperinsulinemia develops, followed by obesity and eventually IR in a vicious cycle [41]. For the reasons mentioned above, an understanding of the molecular mechanism that causes IR is essential, and this information plays a significant role in counteracting the epidemic of type 2 diabetes and cardiovascular diseases that are associated with obesity-related IR.

Strong evidence is provided by early familial genetic studies to prove a genetic basis for both IR and the different constituents of the metabolic syndrome [44–50]. The major part of caveolae is CAV1, which has been revealed to lead to IR and cardiometabolic disease [51]. In pancreatic β-cells, CAV1 plays a role in insulin receptor-mediated signaling, insulin secretion, and probably in diabetes. In physiological low glucose circumstances, CAV1 forms a complex with insulin granule proteins, glucose stimulus mediates CAV1 dissociation and complex disassembly and promotes insulin secretion [52]. On the other side, different evidence indicates a vital role of caveolae in regulating not only insulin secretion but also insulin signaling [53]. Structural studies revealed that in the cell membrane, the insulin receptor is primarily localized in caveolae and only very few receptors are localized outside of caveolae [22]. Impairment of insulin receptor signaling in β cells of the pancreas is observed in either cholesterol depletion or by a mutation that produces a dominant-negative *CAV1*. This issue again highlights the principal role of *CAV1* for the appropriate insulin response.

Moreover, the G32124A (rs3807992) polymorphism is located at the intronic region of the *CAV1* gene. The variation of G32124A intronic polymorphism from *CAV1* may alter the normal expression or protein function of the *CAV1* gene by regulating mRNA [54]. In the *CAV1* genotype, body fat distribution and dyslipidemia are suggested to be caused via the probable mechanism of disruption in insulin signaling [55]. The expression of the *CAV1* gene, in the adipose tissue of obese women who have more fat storage, is greater compared to lean people who have less fat storage [56]. For this reason, we hypothesized that the observed phenotype in minor allele carriers may be manipulated by disruption of insulin receptor function, at least in theory.

A probable role for *CAV1* in metabolic diseases is shown by animal studies and has indicated that *CAV1*-deficient mice exhibit variations in lipid parameters including TC and HDL-C [57]. Moreover, human studies on nonsense mutations show that severe *CAV1* mutations exhibit IR and dyslipidemia [51]. The key mediator of cholesterol homeostasis is *CAV1*, and the function of *CAV1* in HDL-C metabolism was confirmed by higher levels of plasma HDL-C in *CAV1* deficiency [58]. The relationship of *CAV1* variant and dyslipidemia is established by genome-wide association studies (GWAS) that exhibit a link of *CAV1* gene proximal regions to low HDL-C level [29, 59, 60]. Regulation of insulin signaling in adipose tissue could be considered a potential mechanism by which *CAV1* possibly alters lipid metabolism. Insulin promotes lipogenesis and inhibits lipolysis, which finally could alter adipose tissue metabolism. IR is the consequence of obesity which is seen in *CAV1* knock-out mice [61] and is consistent with the medical descriptions of overweight and obese subjects by showing decreased insulin sensitivity in adipose tissues.

HOMA-IR levels are associated with lower HDL-C and higher TC, VLF, FMI levels only in minor allele carriers that possibly could be accompanying with altered CAV1 expression. Our results support the theory that the mechanistic findings in the animal study likely also be valid to humans [62].

To the best of our knowledge, this report is the first study showing that *CAV1* minor allele predicted an association between IR and dyslipidemia and body fat composition. Novel mechanisms such as IR that could be related to specific cardiometabolic disorder pathways associated with *CAV1* deficiency in the human study are explored by this work. Whereas the history of human genetic researches on caveolin is limited, this amount of research confirms a potential association between IR and *CAV1*. Our data have significant clinical consequences. First, we determined a genetic marker that could be used to screen for metabolic disease risk. Second, our results support the hypothesis that *CAV1* is an emerging pathway that IR in humans leads to cardiometabolic disease.

## Conclusion

Based on the present findings, it could be hypothesized that *CAV1* (rs3807992) may be associated with increased metabolic disease risk factors in overweight and obese women. It appears that insulin pathways account for the association between *CAV1* rs3807992 and metabolic factors among minor allele carriers, and this could be critical for clinical diagnosis and gene therapy. Due to limited studies on the *CAV1* polymorphism, more researches are warranted to evaluate the impacts of insulin pathways on caveolin-related metabolic disease.

### Limitations

To the best of our knowledge, this was the first study to investigate the *CAV-1* and IR-pathway with cardiometabolic factors, however, our study had several limitations such as having only overweight and obese women and because of financial limitation we could not perform western blot analysis. Besides, in our study the *CAV1* variant was assessed only in Iranian women, these findings may not be applicable for people of other races. In future studies, investigating samples from a greater geographic area might prove more important findings.

## Data Availability

The data are not publicly available due to containing private information of participants. Data are however available from the authors upon reasonable request and with permission of Khadijeh Mirzaei.

## Abbreviations

BMI: Body mass index
FPG: Fasting plasma glucose
HDL-C: High-density lipoprotein cholesterol
LDL-C: Low-density lipoprotein cholesterol
RFLP: Restriction fragment length polymorphism
TC: Total cholesterol
TG: Triglyceride
FMI: Fat mass index
VFL: Visceral fat level
HOMA: Homeostatic model assessment
